# Delineating tesamorelin response pathways in HIV-associated NAFLD using a targeted proteomic and transcriptomic approach

**DOI:** 10.1038/s41598-021-89966-y

**Published:** 2021-05-18

**Authors:** Lindsay T. Fourman, Takara L. Stanley, James M. Billingsley, Shannan J. Ho Sui, Meghan N. Feldpausch, Autumn Boutin, Isabel Zheng, Colin M. McClure, Kathleen E. Corey, Martin Torriani, David E. Kleiner, Colleen M. Hadigan, Raymond T. Chung, Steven K. Grinspoon

**Affiliations:** 1 Metabolism Unit, Massachusetts General Hospital and Harvard Medical School, 55 Fruit Street 5LON207, Boston, MA 02114 USA; 2 Harvard Chan Bioinformatics Core, Department of Biostatistics, Harvard School of Public Health, Boston, MA 02115 USA; 3 Liver Center, Gastroenterology Division, Massachusetts General Hospital and Harvard Medical School, Boston, MA 02114 USA; 4 Department of Radiology, Massachusetts General Hospital and Harvard Medical School, Boston, MA 02114 USA; 5 Laboratory of Pathology, Center for Cancer Research, National Cancer Institute, Bethesda, MD 20892 USA; 6 National Institute of Allergy and Infectious Diseases, National Institutes of Health, Bethesda, MD 20892 USA

**Keywords:** Non-alcoholic fatty liver disease, HIV infections

## Abstract

NAFLD is a leading comorbidity in HIV with an exaggerated course compared to the general population. Tesamorelin has been demonstrated to reduce liver fat and prevent fibrosis progression in HIV-associated NAFLD. We further showed that tesamorelin downregulated hepatic gene sets involved in inflammation, tissue repair, and cell division. Nonetheless, effects of tesamorelin on individual plasma proteins pertaining to these pathways are not known. Leveraging our prior randomized-controlled trial and transcriptomic approach, we performed a focused assessment of 9 plasma proteins corresponding to top leading edge genes within differentially modulated gene sets. Tesamorelin led to significant reductions in vascular endothelial growth factor A (VEGFA, log_2_-fold change − 0.20 ± 0.35 vs. 0.05 ± 0.34, *P* = 0.02), transforming growth factor beta 1 (TGFB1, − 0.35 ± 0.56 vs. − 0.05 ± 0.43, *P* = 0.05), and macrophage colony stimulating factor 1 (CSF1, − 0.17 ± 0.21 vs. 0.02 ± 0.20, *P* = 0.004) versus placebo. Among tesamorelin-treated participants, reductions in plasma VEGFA (*r* = 0.62, *P* = 0.006) and CSF1 (*r* = 0.50, *P* = 0.04) correlated with a decline in NAFLD activity score. Decreases in TGFB1 (*r* = 0.61, *P* = 0.009) and CSF1 (*r* = 0.64, *P* = 0.006) were associated with reduced gene-level fibrosis score. Tesamorelin suppressed key angiogenic, fibrogenic, and pro-inflammatory mediators. CSF1, a regulator of monocyte recruitment and activation, may serve as an innovative therapeutic target for NAFLD in HIV.

Clinical Trials Registry Number: NCT02196831

## Introduction

In the setting of the modern obesity epidemic, nonalcoholic fatty liver disease (NAFLD) has become a growing threat to public health. NAFLD also is a leading comorbidity in people living with HIV (PLWH)^1^, which may relate to the high prevalence of weight gain and abdominal fat accumulation described among this group^2,3^. Notably, compared to the general population, NAFLD has a more aggressive clinical course with a high frequency of nonalcoholic steatohepatitis (NASH) and fibrosis, and an accelerated rate of fibrosis progression among PLWH^4,5^. Despite the critical need, there are no approved therapies for NAFLD in HIV or the general population^6^.

Tesamorelin, a hypothalamic growth hormone-releasing hormone (GHRH) analogue that augments endogenous pulsatile growth hormone (GH) and insulin-like growth factor 1 (IGF-1) secretion, is FDA-approved for the treatment of abdominal fat accumulation in HIV. In a recent randomized placebo-controlled trial, we examined the effects of tesamorelin on liver fat and histology among PLWH with NAFLD. In this prior study, tesamorelin was found to significantly reduce liver fat and prevent fibrosis progression over one year^7^. We further performed a transcriptomic analysis on paired liver biopsy specimens from this trial to identify key hepatic pathways that were differentially modulated by tesamorelin versus placebo. Using gene set enrichment analysis (GSEA) of hallmark gene sets, we found that tesamorelin led to hepatic upregulation of gene sets involved in oxidative phosphorylation, and downregulation of gene sets pertaining to inflammation, tissue repair, and cell division^8^.

Leveraging these results, we have now performed a focused proteomic analysis of top leading edge genes in key pathways flagged by our transcriptomic analysis. For each protein, we compared changes in protein levels by treatment status, and assessed associations of such changes with radiographic, histologic, and transcriptomic indices of NAFLD phenotype. Using this overlaid transcriptomic and proteomic approach, we identified novel effects of tesamorelin to reduce 3 plasma proteins in proportion to a decline in NAFLD severity. The current study deepens our understanding of the biologic effects of GH axis augmentation among PLWH with NAFLD and introduces innovative potential therapeutic targets and protein signatures of treatment response that warrant further investigation.

## Methods

### Study design

We previously conducted a randomized, double blind trial in which PLWH with NAFLD were assigned to receive the GHRH analogue tesamorelin 2 mg daily or identical placebo for 12 months^7^. Utilizing plasma specimens from this trial, the current study builds significantly on prior, purely transcriptomic analyses^8^ to examine specific proteins corresponding to top leading edge genes in pathways responsive to tesamorelin. Specifically, we investigated changes in circulating levels of these proteins between treatment groups and assessed for the first time relationships of these proteins to histologic, radiographic, and transcriptomic indices to elucidate potential mechanisms of tesamorelin response.

We enrolled 61 men and women 18–70 years old who had documented HIV infection and liver steatosis as defined by hepatic fat fraction ≥ 5% on ^1^H-magnetic resonance spectroscopy (^1^H-MRS). Participants were required to have been on stable antiretroviral therapy (ART) for ≥ 3 months with CD4^+^ T cell count > 100 cells/mm^3^ and HIV viral load < 400 copies/mL. Exclusion criteria included excess alcohol use (> 20 g daily for women or > 30 g daily for men), active hepatitis B or C, other known hepatic disease, cirrhosis, and inadequately controlled diabetes mellitus (HbA1c ≥ 7%)^7^. Participants were enrolled at the Massachusetts General Hospital (MGH, Boston, MA) and the National Institutes of Health (NIH, Bethesda, MD) between August 20, 2015 and January 16, 2019. Informed consent in writing was obtained from each participant. The institutional review boards at MGH and NIH approved this study. All methods were carried out in accordance with guidelines and regulations.

### Study procedures

Study procedures for the parent clinical trial have been described in detail elsewhere^7^. All study procedures were conducted in a fasting state. In brief, hepatic ^1^H-MRS was performed for measurement of hepatic fat fraction at baseline and 12 months. An ultrasound-guided percutaneous liver biopsy yielding two cores also was completed at each time point. The first core was fixed in formalin, and subsequently underwent histopathologic review by a single expert pathologist blinded to treatment (D.E.K., National Institutes of Health). Histological scoring, including NAFLD Activity Score (NAS) and fibrosis stage, was performed according to the Nonalcoholic Steatohepatitis Clinical Research Network scoring system^9^. The second core was placed in an RNA stabilization reagent (RNAlater, Qiagen) and stored at − 80 °C for gene expression analyses. Blood specimens were collected at baseline and 12 months and stored at − 80 °C. Serum IGF-1 was measured using standard techniques (Quest Laboratories).

### Hepatic transcriptomic assessment

Liver tissue underwent RNA extraction, cDNA library construction, and Illumina sequencing using methods that have been previously described^8^. To identify pathways differentially modulated from pre- to post-treatment time points between tesamorelin- and placebo-treated participants, GSEA was performed using the desktop module from the Broad Institute (www.broadinstitute.org/gsea/). Gene sets used corresponded to the Molecular Signatures Database (MsigDB) hallmark gene set collection^10^. GSEA leading edge genes were the subset of genes in a significantly enriched gene set that accounted for the enrichment signal and were used for the subsequent quantification of pathway gene expression. Gene sets with false discovery rate (FDR) < 0.05 were considered enriched.

**Figure 1:**
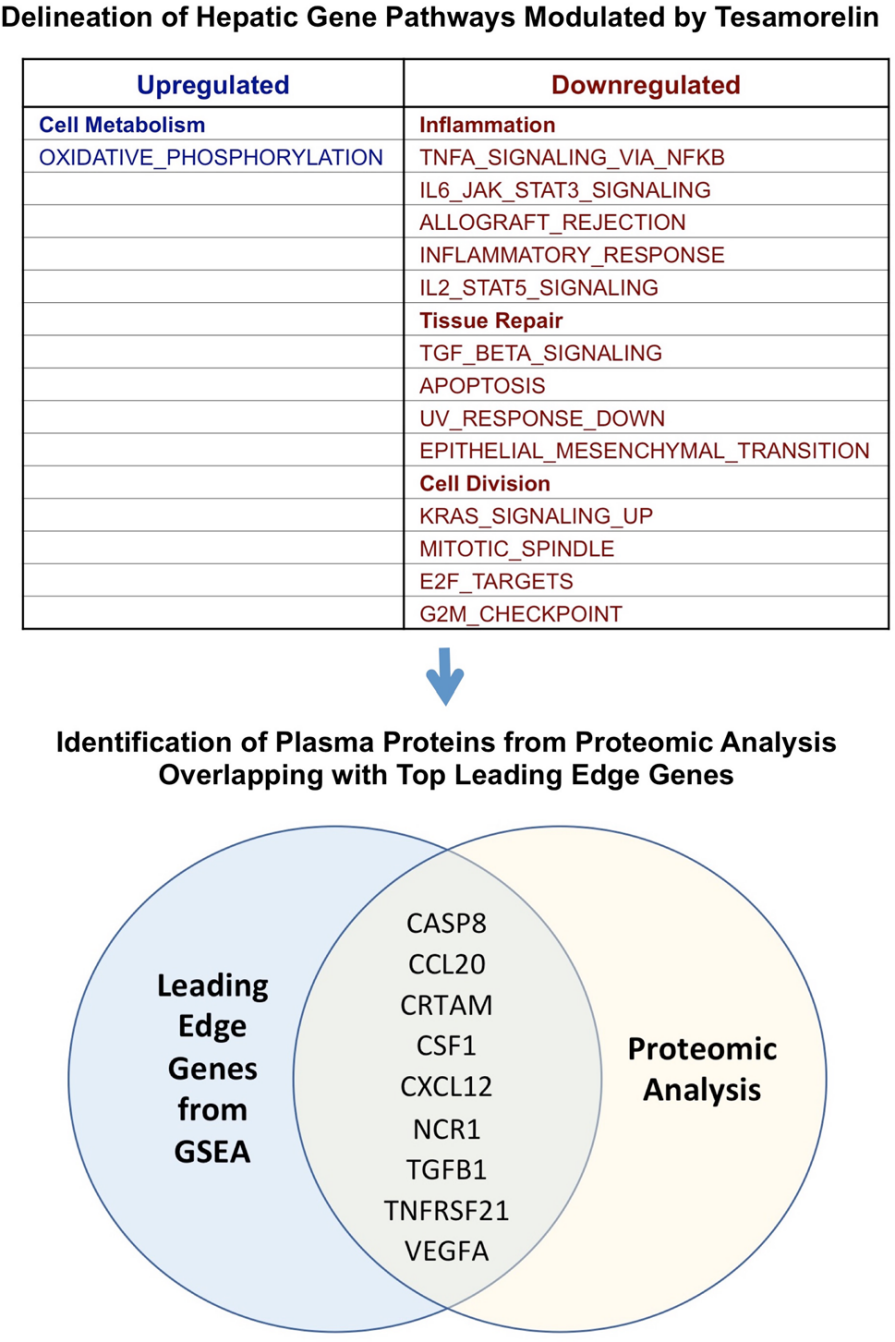
Analysis Schema. A total of 9 plasma proteins were examined, corresponding to top leading edge genes within hepatic gene pathways differentially modulated by tesamorelin. Abbreviations: CASP8, caspase 8; CCL20, C–C motif chemokine ligand 20; CRTAM, cytotoxic and regulatory T-cell molecule; CSF1, macrophage colony stimulating factor 1; CXCL12, C-X-C motif chemokine ligand 12; NCR1, natural cytotoxicity triggering receptor 1; TGFB1, transforming growth factor beta 1; TNFRSF21, tumor necrosis factor receptor superfamily member 21; VEGFA, vascular endothelial growth factor A.

Utilizing this approach, we previously discovered 14 hallmark gene pathways that were differentially regulated by tesamorelin versus placebo. In this regard, a gene set pertaining to oxidative phosphorylation was upregulated with treatment. Furthermore, 13 gene sets involved in inflammation, tissue repair, and cell division were downregulated among tesamorelin-treated individuals (Fig. 1)^8^. The RNA-Seq data were submitted to the Gene Expression Omnibus repository at the National Center for Biotechnology Information (accession number GSE150026).

### Plasma proteomic assessment

For this analysis, we assessed change over 12 months in targeted proteins using an Olink Multiplex proximity extension assay (PEA) platform. The PEA is an affinity-based assay that characterizes abundance levels of pre-determined sets of proteins. Each protein is targeted by a unique pair of oligonucleotide-labeled antibodies. When in close proximity, the oligonucleotides undergo a proximity-dependent DNA polymerization event to form a PCR target sequence. The resultant DNA sequence is detected and quantified using standard real-time PCR on the Fluidigm BioMark HD real-time PCR platform. The PEA gives protein abundance levels of Normalized Protein eXpression (NPX) on a log_2_-scale. Assay characteristics including detection limits and measurements of assay performance are available from the manufacturer (www.olink.com). Specificity is high due to the precision of the methodology, which enabled assessment of change over time. Across all proteins within the high-multiplex panel utilized (below), the mean intra-assay and inter-assay variation were reported as 8.3% and 11.5%, respectively.

### Targeted proteomic analysis

The objective of the current study was to delineate potential response pathways of tesamorelin effects in NAFLD. We also sought to determine a protein signature that might be used to detect a treatment response to tesamorelin among patients with NAFLD. To do so, we flagged all plasma proteins within a high-multiplex panel of nearly 100 proteins (Olink Immuno-Oncology; see www.olink.com for the complete protein list) that were found to overlap with top leading genes from tesamorelin-responsive gene sets^8^. Among this targeted set of proteins, we compared changes in plasma levels by treatment status. Proteins found to be differentially modulated by tesamorelin relative to placebo were then examined in relation to radiographic, histologic, and transcriptomic indices of NAFLD severity both at baseline and longitudinally. As a surrogate for fibrosis stage, we utilized a gene-level fibrosis score derived from the hepatic expression of 18 genes shown to correlate with fibrosis^11^, which was validated in our current sample to histological changes as we have previously described^8^. We also related changes in levels of these proteins to changes in their corresponding hepatic transcript level and change in serum IGF-1.

Continuous variables were expressed as mean ± standard deviation, whereas categorical variables were indicated as a frequency (%). Differences between groups were compared using a two-tailed independent samples *t*-test for continuous variables and chi-square test for categorical variables. Correlations were assessed with Pearson correlation coefficient. A critical value of *P* ≤ 0.05 was the pre-defined threshold for statistical significance. Statistical analyses were performed using JMP Pro 14 (SAS Institute Inc., Cary, North Carolina, USA).

## Results

### Characteristics of study participants

Of 61 participants with HIV-associated NAFLD in the randomized-controlled trial, 58 individuals had a plasma protein panel obtained at baseline that was available for analysis. Moreover, 44 of these individuals (20 assigned to tesamorelin, 24 assigned to placebo) had a plasma protein panel repeated at 12 months. Characteristics of each treatment group are summarized in Supplementary Table 1 and have been described elsewhere^7^. Tesamorelin and placebo groups were well balanced with respect to key clinical variables. Furthermore, participants with paired data were comparable to the overall sample. Briefly, participants (53 ± 7 years old, 79% male) had well-controlled HIV infection for 17 ± 9 years. All subjects received stable ART. Baseline hepatic fat content was 14 ± 8% as measured by hepatic ^1^H-MRS. A total of 33% and 43% had histologic evidence of NASH and fibrosis, respectively, on initial liver biopsy.

### Plasma proteins differentially regulated by tesamorelin

**Figure 2:**
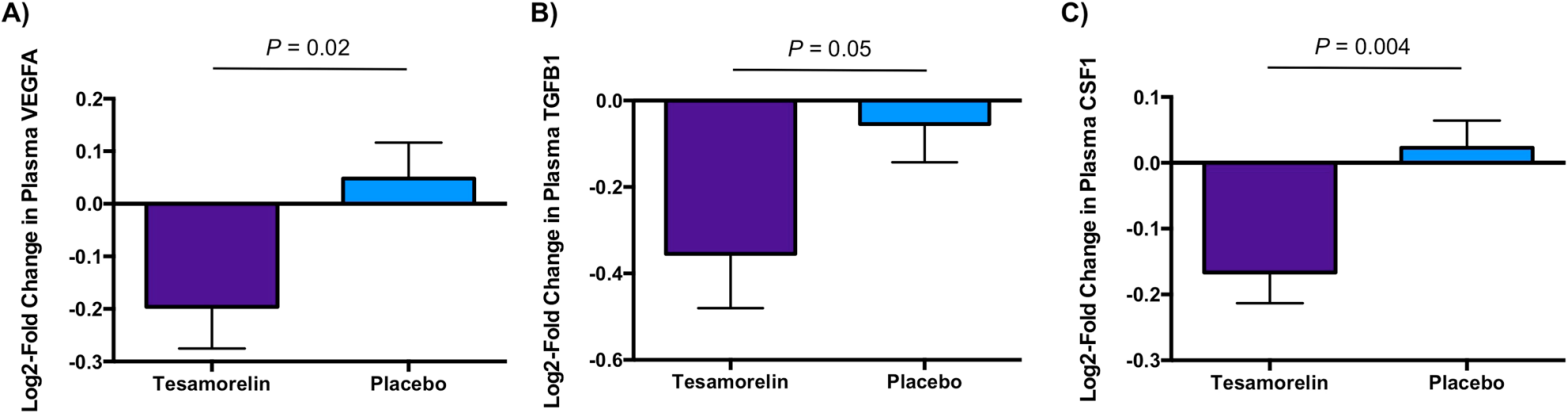
Differential Changes in Plasma VEGFA, TGFB1, and CSF1 by Treatment Status. Tesamorelin led to significant reductions in plasma **(A)** VEGFA (log_2_-fold change, mean ± SD, − 0.20 ± 0.35 vs. 0.05 ± 0.34, *P* = 0.02), **(B)** TGFB1 (log_2_-fold change − 0.35 ± 0.56 vs. − 0.05 ± 0.43, *P* = 0.05), and **(C)** CSF1 (log_2_-fold change − 0.17 ± 0.21 vs. 0.02 ± 0.20, *P* = 0.004) compared to placebo. Bars and error bars indicate mean and standard error of the mean, respectively. *Abbreviations:* CSF1, macrophage colony stimulating factor 1; TGFB1, transforming growth factor beta 1; VEGFA, vascular endothelial growth factor A.

Nine plasma proteins were identified as corresponding to top leading edge genes modulated by tesamorelin (Fig. 1). These leading edge genes were contained within gene sets pertaining to inflammation, tissue repair, and cell division that were downregulated by treatment with tesamorelin versus placebo (Table 1). Of these proteins, treatment with tesamorelin led to reductions in plasma VEGFA (log_2_-fold change − 0.20 ± 0.35 vs. 0.05 ± 0.34, *P* = 0.02), TGFB1 (log_2_-fold change − 0.35 ± 0.56 vs. − 0.05 ± 0.43, *P* = 0.05), and CSF1 (log_2_-fold change − 0.17 ± 0.21 vs. 0.02 ± 0.20, *P* = 0.004) compared to placebo (Fig. 2). Moreover, plasma CCL20 tended to decrease with tesamorelin though the difference between groups did not reach statistical significance (log_2_-fold change − 0.28 ± 0.88 vs. 0.20 ± 0.79, *P* = 0.06). The effect of treatment versus placebo on all 9 plasma proteins is summarized in Supplementary Table 2.

**Table 1:** Overlap of plasma proteins studied with top leading edge genes within differentially modulated gene sets.

Plasma protein	Gene set with corresponding top leading edge gene
CASP8	APOPTOSIS
CCL20	INFLAMMATORY_RESPONSE, KRAS_SIGNALING_UP, TNFA_SIGNALING_VIA_NFKB
CRTAM	ALLOGRAFT_REJECTION
CSF1	IL6_JAK_STAT3_SIGNALING, INFLAMMATORY_RESPONSE
CXCL12	EPITHELIAL_MESENCHYMAL_TRANSITION
NCR1	ALLOGRAFT_REJECTION
TGFB1	TGF_BETA_SIGNALING, IL6_JAK_STAT3_SIGNALING
TNFRSF21	IL6_JAK_STAT3_SIGNALING, IL2_STAT5_SIGNALING
VEGFA	TNFA_SIGNALING_VIA_NFKB

Abbreviations: CASP8, caspase 8; CCL20, C–C motif chemokine ligand 20; CRTAM, cytotoxic and regulatory T-cell molecule; CSF1, macrophage colony stimulating factor 1; CXCL12, C-X-C motif chemokine ligand 12; NCR1, natural cytotoxicity triggering receptor 1; TGFB1, transforming growth factor beta 1; TNFRSF21, tumor necrosis factor receptor superfamily member 21; VEGFA, vascular endothelial growth factor A.

### Association of key plasma proteins with NAFLD phenotype

Given their differential regulation by tesamorelin, we next studied relationships of VEGFA, TGFB1, and CSF1 with NAFLD phenotype. At baseline, in the overall sample, plasma CSF1 level directly correlated with NAS score (*r* = 0.38, *P* = 0.004) and gene-level fibrosis score (*r* = 0.37, *P* = 0.03). In contrast, VEGFA and TGFB1 were not found to be associated with either of these parameters. Furthermore, there was no baseline relationship of VEGFA, TGFB1, or CSF1 with hepatic fat fraction.

**Figure 3:**
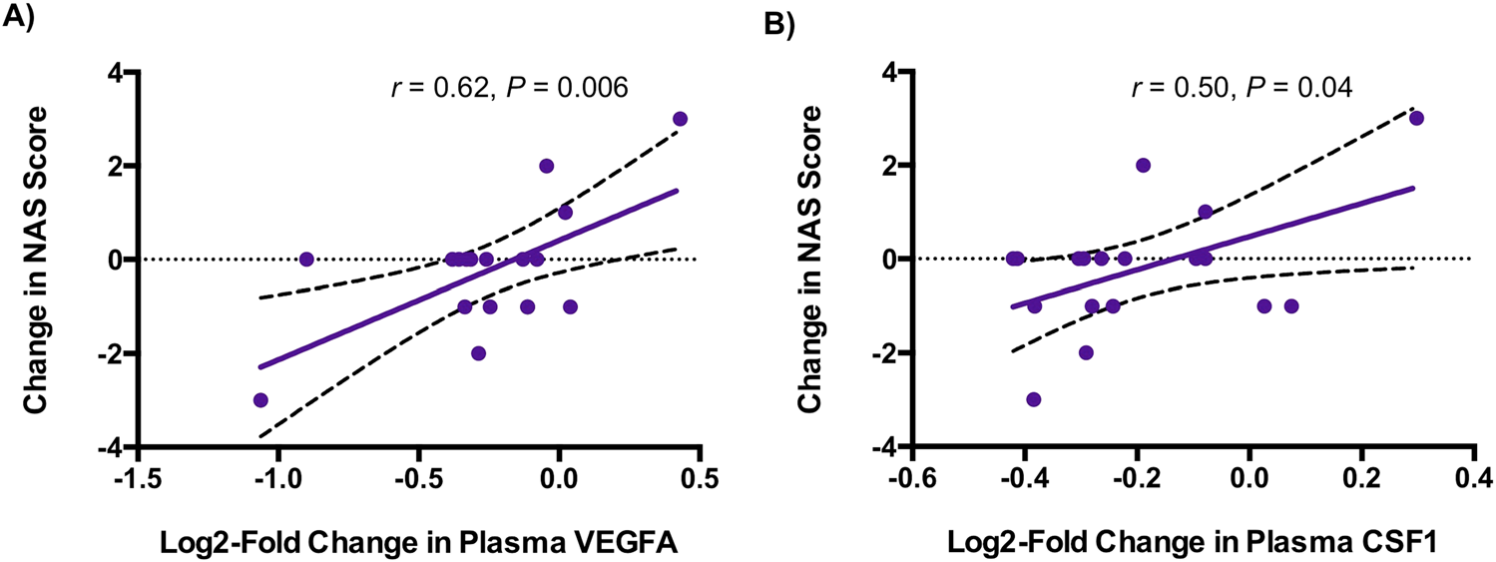
Relationship of Changes in Plasma VEGFA and CSF1 with Change in NAS Score in Tesamorelin-Treated Participants. Within the tesamorelin-treated arm, reductions in plasma **(A)** VEGFA (*r* = 0.62, *P* = 0.006) and **(B)** CSF1 (*r* = 0.50, *P* = 0.04) were associated with a decrease in NAS score. Linear regression lines with 95% confidence intervals are shown. *Abbreviations:* CSF1, macrophage colony stimulating factor 1; NAS, NAFLD activity score; VEGFA, vascular endothelial growth factor A.

**Figure 4:**
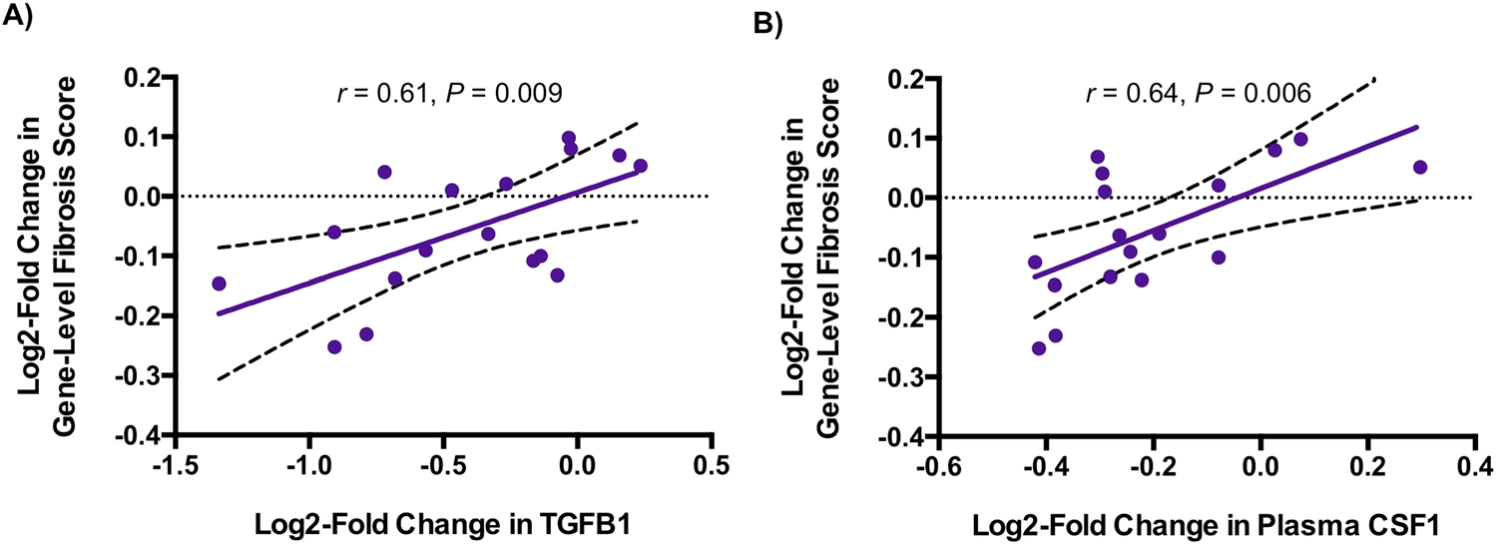
Relationship of Changes in Plasma TGFB1 and CSF1 with Change in Gene-Level Fibrosis Score in Tesamorelin-Treated Participants. Among tesamorelin-treated participants, declines in plasma (**A)** TGFB1 (r = 0.61, *P* = 0.009) and **(B)** CSF1 (*r* = 0.64, *P* = 0.006) were associated with improved gene-level fibrosis score. Linear regression lines with 95% confidence intervals are shown. *Abbreviations:* CSF1, macrophage colony stimulating factor 1; TGFB1, transforming growth factor beta 1.

Within the tesamorelin-treated arm, reductions in plasma VEGFA (*r* = 0.62, *P* = 0.006) and CSF1 (*r* = 0.50, *P* = 0.04) correlated with a decline in NAS score (Fig. 3, Supplementary Table 3). Furthermore, among tesamorelin-treated participants, reductions in TGFB1 (*r* = 0.61, *P* = 0.009) and CSF1 (*r* = 0.64, *P* = 0.006) were associated with a decline in gene-level fibrosis score (Fig. 4). Changes in these 3 plasma proteins were not found to correlate with change in NAS score or gene-level fibrosis score among placebo-treated participants, or with change in hepatic fat fraction among either treatment group.

### Association of changes in key plasma proteins with changes in hepatic transcript levels and serum IGF-1

To elucidate the regulation of plasma VEGFA, TGFB1, and CSF1, we next investigated their relationships with corresponding hepatic transcript levels and serum IGF-1 levels within the overall sample. Only CSF1 exhibited a correlation between changes in plasma protein and hepatic transcript levels (*r* = 0.50, *P* = 0.002). Additionally, an increase in serum IGF-1 was associated with a linear decline in CSF1 (*r* = − 0.38, *P* = 0.01), whereas analogous relationships were not observed with VEGFA or TGFB1.

## Discussion

In the current study, elaborating upon a prior hepatic transcriptomic analysis, we examined effects of tesamorelin on key plasma proteins in PLWH with NAFLD. These proteins were selected from an immunologic high-multiplex panel as corresponding to top leading edge genes within pathways differentially modulated by treatment versus placebo. Through this targeted approach, we demonstrated novel effects of tesamorelin to reduce plasma levels of 3 critical proteins whose transcript levels similarly were shown to decline in the liver compared to placebo: vascular endothelial growth factor A (VEGFA), transforming growth factor beta 1 (TGFB1), and macrophage colony stimulating factor 1 (CSF1). Notably, among tesamorelin-treated participants, decreases in plasma levels of VEGFA and CSF1 correlated with a reduction in NAS score, whereas declines in TGFB1 and CSF1 were associated with a decrease in fibrosis-related gene score. These findings deepen our knowledge of the biologic actions of GH axis augmentation among PLWH with NAFLD, demonstrating suppression of key circulating angiogenic, fibrogenic, and pro-inflammatory mediators, with evidence linking tesamorelin-mediated changes in these pathways to improvements in key histologic and transcriptomic indices. This work also uncovers potential biomarkers of a treatment response to tesamorelin as well as novel targets for future drug discovery.

In this study, we found that tesamorelin decreased plasma levels of VEGFA relative to placebo. Moreover, tesamorelin-treated participants who had the largest reduction in VEGFA demonstrated the greatest decline in NAS score. Angiogenesis plays a pivotal role in the pathogenesis of NAFLD. In this regard, liver biopsies from patients with NASH were found to exhibit significant neovascularization in proportion to the extent of fibrosis, whereas minimal neovascularization was observed in cases of simple steatosis^12^. VEGFA is a potent angiogenic factor that stimulates the proliferation, migration, and differentiation of endothelial cells. Studies have shown increased circulating levels^13^ and hepatic upregulation^14^ of VEGF in patients with NAFLD or NASH compared to controls. Conversely, blockade of VEGF receptor 2 was demonstrated to prevent and to treat hepatic steatosis and hepatocyte ballooning in mouse models of NASH^15^. Given the role of VEGFA in NAFLD pathogenesis, the reduction in this angiogenic factor by tesamorelin may in part underlie its attenuation of NAFLD progression in HIV. Whether VEGFA may serve as a biomarker in this regard merits further exploration in future studies.

We additionally demonstrated that tesamorelin decreased plasma levels of TGFB1 as compared to placebo among PLWH with NAFLD. Moreover, a decline in TGFB1 corresponded to a reduction in fibrosis-related gene score within the tesamorelin-treated arm. TGFB1 is a pleiotropic cytokine that is integral to the regulation of various cellular processes including proliferation, differentiation, migration, and death^16^. Human and animal studies have shown hepatic upregulation of *TGFB1* expression with NASH and fibrosis^17,18^. Moreover, in a NASH mouse model, hepatocyte-specific TGFB receptor type II (*Tgfbr2*) deficiency reduced hepatic steatosis, inflammation, hepatic stellate cell activation, and collagen deposition^17^. Given the role of TGFB1 in NAFLD, the decline in this growth factor with tesamorelin may contribute to its attenuation of NAFLD, and particularly fibrosis, progression in PLWH.

Importantly, we found that tesamorelin decreased plasma CSF1 relative to placebo among PLWH with NAFLD. Furthermore, baseline plasma CSF1 level directly related to NAS score and gene-level fibrosis score among our overall sample, whereas tesamorelin-treated individuals with the greatest reduction in plasma CSF1 had the most marked decline in NAS score and gene-level fibrosis score. CSF1 is a cytokine and hematopoietic growth factor that regulates the differentiation, migration, proliferation, function, and survival of macrophages that belong to the mononuclear phagocyte system^19^. In the liver, this protein plays an integral role in the regulation of Kupffer cells^20^. Accordingly, osteopetrotic (*Csf1*^*op*^*/Csf1*^*op*^) mice deficient in functional CSF1 exhibited a 30% decline in Kupffer cells and marked reductions in other tissue-resident macrophage populations as compared to wild-type littermates^21^. Similarly, treatment of macaques with a neutralizing monoclonal antibody to CSF1 led to a two- to threefold decrement in Kupffer cell count^22^.

While hepatic expression of *CSF1* has been previously shown to be upregulated in NASH^14^, to our knowledge, no prior study has linked a decline in CSF1 with an improvement in NAFLD severity. Nonetheless, given that Kupffer cells are key players in NAFLD initiation and progression^23,24^, it is plausible that a strategy that blunts the activity of this cell population would attenuate the course of disease. Notably, while CSF1 receptor antagonists have been studied with great interest in the context of various inflammatory disorders^19^, their use in chronic liver disease has yet to be explored. Intriguingly, in addition to its intrahepatic effects, systemic attenuation of CSF1 signaling may ameliorate cardiovascular disease to which PLWH with NAFLD are prone. Previous studies have revealed the presence of CSF1 protein in coronary plaque^25^, whereas circulating CSF1 levels predicted major adverse cardiac events in a Mendelian randomization analysis^26^. Taken together, these data suggest the potential importance of CSF1 in PLWH, and highlight the need for further studies of tesamorelin effects on this protein pathway among this patient population.

This study has some limitations but a number of strengths. To our knowledge, this is the first study to employ a dual transcriptomic and proteomic approach to identify novel circulating protein signatures and therapeutic targets for NAFLD in PLWH. In addition, these results are demonstrated in a randomized trial with the presence of a placebo comparator group, and the availability of detailed phenotypic data on study participants. Given that subjects had well-controlled HIV infection on modern ART, our findings are also broadly generalizable to the large proportion of PLWH with NAFLD. Our analysis detected effect sizes of approximately 15–25% in key proteins between treatment and placebo groups. The biological significance of these changes was supported by their relationships with histological indices over time in a randomized-controlled trial. Nonetheless, the effects on critical hepatic pathways identified in this analysis require further investigation in future large-scale studies. While the changes in these proteins related significantly to changes in histological indices of NAFLD/NASH, circulating levels may also reflect direct effects of tesamorelin on circulating immune cells, such as macrophages, or effects on other tissues, which require further study.

Taken together, in this focused proteomic analysis guided by a whole transcriptomic approach, we identified VEGFA, TGFB1, and CSF1 as novel proteins whose circulating levels were reduced by tesamorelin in association with a decline in NAFLD severity among PLWH with NAFLD. The current study extends our knowledge of the biologic actions of GH axis augmentation to encompass systemic changes in key angiogenic, fibrogenic, and pro-inflammatory factors that may underlie a tesamorelin response. Notably, among these proteins, CSF1 is a key regulator of monocyte recruitment and activation that has not been previously well studied in the context of NAFLD, but may serve as an innovative therapeutic target in this regard. Lastly, our data indicate that VEGFA, TGFB1, and CSF1 are candidate biomarkers for a tesamorelin response among PLWH with NAFLD, which should be explored in future studies.

## Supplementary Information

Supplementary Information.
