# Supplementary material for: Delineating tesamorelin response pathways in HIV-associated NAFLD using a targeted proteomic and transcriptomic approach
Source: Sci Rep. 2021 May 18;11:10485. doi: 10.1038/s41598-021-89966-y (PMC8131688; doi:10.1038/s41598-021-89966-y)
Supplement: Supplementary file 1 — Supplementary Information. [file 41598_2021_89966_MOESM1_ESM.pdf]

Supplementary Table 1: Baseline Demographic and Clinical Characteristics

	Overall Sample		Subset with Paired Protein Panels	
	**Tesamorelin** (n = 31)	**Placebo** (n = 30)	**Tesamorelin** (n = 20)	**Placebo** (n = 24)
Age (years)	52 ± 8	54 ± 7	53 ± 6	54 ± 7
% Male	77	80	70	79
% Race				
White	68	63	55	71
Black	26	33	40	29
Other	6	3	5	0
% Hispanic	19	10	10	8
Duration of HIV infection (years)	16 ± 9	18 ± 8	17 ± 10	18 ± 8
CD4 count (cells/mm^3)	733 ± 290	798 ± 260	740 ± 265	792 ± 269
log HIV viral load	0.34 ± 0.59	0.50 ± 0.74	0.39 ± 0.61	0.41 ± 0.66
% Current Antiretroviral Use				
NRTI	87	97	85	96
NNRTI	39	37	30	38
PI	29	20	25	25
Integrase Inhibitor	68	60	80	58
% Type 2 Diabetes	13	13	5	13
Liver biopsy length (mm)	13 ± 3	13 ± 3	14 ± 2	13 ± 3
Hepatic Fat Fraction (%)	13 ± 8	15 ± 9	15 ± 9	15 ± 9
% NASH	34	31	22	35
% Fibrosis	48	38	39	39
Stage 1	14	17	17	13
Stage 2	21	14	17	17
Stage 3	14	7	6	9

There were no statistically significant differences between treatment groups at baseline for any of the variables shown above.

Continuous variables are presented as mean ± standard deviation.

Abbreviations: NASH, nonalcoholic steatohepatitis; NNRTI, non-nucleoside reverse transcriptase inhibitor; NRTI, nucleoside reverse transcriptase inhibitor; PI, protease inhibitor.

Supplementary Table 2: Effects of Tesamorelin Versus Placebo on Select Plasma Proteins

Plasma Protein	Log_2-Baseline		Log_2-Fold Change		*P*-value
	**Tesamorelin** (n = 20)	**Placebo** (n = 24)	**Tesamorelin** (n = 20)	**Placebo** (n = 24)	
CASP8	4.91 (4.52, 5.30)	4.76 (4.39, 5.13)	-0.37 ± 0.72	-0.02 ± 0.65	0.10
CCL20	7.53 (6.85, 8.21)	7.15 (6.62, 7.68)	-0.28 ± 0.88	0.20 ± 0.79	0.06
CRTAM	5.71 (5.40, 6.02)	6.08 (5.89, 6.27)	-0.35 ± 0.73	-0.04 ± 0.50	0.10
CSF1	9.84 (9.77, 9.92)	9.79 (9.72, 9.85)	-0.17 ± 0.21	0.02 ± 0.20	**0.004**
CXCL12	1.16 (1.08, 1.25)	1.15 (1.10, 1.20)	-0.03 ± 0.18	-0.003 ± 0.17	0.54
NCR1	3.05 (2.81, 3.29)	3.21 (2.99, 3.42)	-0.20 ± 0.52	-0.08 ± 0.31	0.37
TGFB1	8.99 (8.73, 9.24)	8.82 (8.67, 8.98)	-0.35 ± 0.56	-0.05 ± 0.43	**0.05**
TNFRSF21	7.57 (7.44, 7.71)	7.62 (7.52, 7.72)	-0.13 ± 0.22	-0.05 ± 0.23	0.25
VEGFA	8.13 (7.97, 8.29)	7.92 (7.81, 8.03)	-0.20 ± 0.35	0.05 ± 0.34	**0.02**

Bold text denotes $P < 0.05$. Data are represented as mean (95% confidence interval) or mean ± SD.

Abbreviations: CASP8, caspase 8; CCL20, C-C motif chemokine ligand 20; CRTAM, cytotoxic and regulatory T-cell molecule; CSF1, macrophage colony stimulating factor 1; CXCL12, C-X-C motif chemokine ligand 12; NCR1, natural cytotoxicity triggering receptor 1; TGFB1, transforming growth factor beta 1; TNFRSF21, tumor necrosis factor receptor superfamily member 21; VEGFA, vascular endothelial growth factor A.

Supplementary Table 3: Relationships of Changes in Plasma Proteins Downregulated by Tesamorelin with Change in NAFLD Severity

**Log_2-Fold Change in Plasma Protein**	**Change in NAS Score**		**Change in Hepatic Fat Fraction (%)**		**Change in Fibrosis-Related Gene Score**	
	Tesamorelin (n = 18)	*Placebo* (n = 22)	*Tesamorelin* (n = 19)	*Placebo* (n = 23)	*Tesamorelin* (n = 17)	*Placebo* (n = 19)
VEGFA	*r* = 0.62, ***P* = 0.006**	*r* = 0.11, *P* = 0.62	*r* = 0.30, *P* = 0.22	*r* = 0.12, *P* = 0.57	*r* = 0.30, *P* = 0.24	*r* = -0.04, *P* = 0.86
TGFB1	*r* = 0.31, *P* = 0.22	*r* = 0.24, *P* = 0.29	*r* = 0.17, *P* = 0.49	*r* = 0.34, *P* = 0.11	*r* = 0.61, ***P* = 0.009**	*r* = -0.07, *P* = 0.79
CSF1	*r* = 0.50, ***P* = 0.04**	*r* = 0.21, *P* = 0.35	*r* = 0.13, *P* = 0.60	*r* = 0.16, *P* = 0.46	*r* = 0.64, ***P* = 0.006**	*r* = 0.29, *P* = 0.23

Bold text denotes $P < 0.05$.

Abbreviations: CSF1, macrophage colony stimulating factor 1; NAS, NAFLD activity score; TGFB1, transforming growth factor beta 1; VEGFA, vascular endothelial growth factor A.
